# Poor Immune Reconstitution in HIV-Infected Patients Associates with High Percentage of Regulatory CD4^+^ T Cells

**DOI:** 10.1371/journal.pone.0057336

**Published:** 2013-02-20

**Authors:** Ana Horta, Claudia Nobrega, Pedro Amorim-Machado, Vitor Coutinho-Teixeira, Palmira Barreira-Silva, Susana Boavida, Patrício Costa, Rui Sarmento-Castro, António Gil Castro, Margarida Correia-Neves

**Affiliations:** 1 Life and Health Sciences Research Institute (ICVS), School of Health Sciences, University of Minho, Braga, Portugal; 2 ICVS/3B's, PT Government Associate Laboratory, Braga/Guimarães, Portugal; 3 Infectious Diseases Service of the Centro Hospitalar do Porto, Hospital Joaquim Urbano Unit, Porto, Portugal; New York University, United States of America

## Abstract

CD4^+^ regulatory T cells (Tregs) are essential for the maintenance of the immune system's equilibrium, by dampening the activation of potential auto-reactive T cells and avoiding excessive immune activation. To correctly perform their function, Tregs must be maintained at the right proportion with respect to effector T cells. Since this equilibrium is frequently disrupted in individuals infected with the human immunodeficiency virus (HIV), we hypothesize that its deregulation could hamper immune reconstitution in patients with poor CD4^+^ T cell recovery under highly active antiretroviral therapy (HAART). We analysed Tregs percentages amongst CD4^+^ T cells in 53 HIV-infected patients under HAART, with suppression of viral replication and distinct levels of immune reconstitution. As controls, 51 healthy individuals were also analysed. We observed that amongst the patients with Nadir values (the lowest CD4^+^ T cell counts achieved) <200 cells/µL, the individuals with high Tregs percentages (≥10% of total CD4^+^ T cells) had the worse CD4^+^ T cell reconstitution. In accordance, the well-described direct correlation between the Nadir value and CD4^+^ T cell reconstitution is clearly more evident in individuals with high Tregs proportions. Furthermore, we observed a strong negative correlation between Tregs percentages and CD4^+^ T cell recovery among immunological non-responder HIV^+^ individuals. All together, this work shows that high Tregs frequency is an important factor associated with sub-optimal CD4^+^ T cell recovery. This is particularly relevant for immunological non-responders with low Nadir values. Our results suggest that the Tregs proportion might be of clinical relevance to define cut-offs for HAART initiation.

## Introduction

Infection with HIV initiates a series of events that ultimately lead to profound immunosuppression, caused by functional abnormalities in the immune system, mainly due to severe depletion of CD4^+^ T cells [1].

The introduction of HAART has led to very important declines in both mortality and morbidity due to HIV infection [2]; however, even though many patients steadily recover their CD4^+^ T cell compartment over several years post-HAART initiation, the degree of immune recovery achieved is highly variable. On this, studies indicate that even after several years of treatment, a proportion of patients (from 15% to 40%) feature abnormally low CD4^+^ T cell counts despite suppression of HIV replication [3], [4], [5], [6]. This group of individuals is referred to as immunological discordants or non-responders and, unlike full responders, they are at increased risk of clinical progression to acquired immunodeficiency syndrome (AIDS)-related and non-related illnesses and death [2].

Sub-optimal CD4^+^ T cell recovery may result from excessive/premature cell death, decreased peripheral proliferation and/or reduced production of these cells by the thymus. Several factors have been suggested to contribute to this limited ability of the CD4^+^ T cell compartment to normalise (reviewed in [7]) such as advanced age [8], low baseline CD4^+^ T cell counts [6], [8], [9], residual HIV replication [10], chronic immune activation [11], abrogated thymic function [12], [13], gender [14], [15] and genetic polymorphisms associated with increased programmed cell death [16], [17]. While all these factors are definitely relevant in establishing different immune reconstitution profiles, there may be other factors also contributing to this process [7].

Tregs are essential for the maintenance of self-tolerance and immune homeostasis [18] and have been widely studied in the context of HIV infection. Most studies have focused on whether or not these cells are directly infected by HIV, to what extent are they depleted/expanded, and their role during the course of disease progression from HIV infection to AIDS. The ability of HIV to directly infect Tregs is still a subject of debate. Whilst it has been reported that they are susceptible to HIV infection *in vitro* [19], [20], other studies showed that exposure of Tregs to HIV selectively promotes their survival via a CD4-gp120–dependent pathway [21]. Moreover, the accumulation of Tregs in the gut or in the tonsils of HAART-naïve HIV^+^ patients also argues against increased killing of these cells in compartments where viral replication is occurring [22], [23].

During the course of disease progression in HAART-naïve patients, Tregs seems to act as a double-edged sword. On one hand frequencies of these cells have been shown to negatively correlate with the levels of immune activation [24], [25], [26], [27], [28], which is one of the key contributors to HIV disease progression [29], [30], [31]; on the other hand, high levels of Tregs have also been linked with suppression of HIV-specific CD4^+^ and CD8^+^ T cell activity and, thus, in this way Tregs could be linked to a worse disease prognosis [32], [33], [34]. Tregs have also been investigated in the context of HAART-associated immune reconstitution, although to a much lesser extent. Results so far also lack a consensus. Some authors found elevated levels of Tregs in aviremic HIV^+^ patients in comparison to healthy controls [35], [36], [37], [38], whilst others described Tregs frequencies as following a “biphasic curve” during the first year of HAART [39], with an initial increase and a subsequent return to levels comparable to controls [22], [23], [39]. The observation that the proportion of Tregs in HAART-treated aviremic HIV^+^ individuals is quite diverse prompted us to explore whether distinct values were associated with differences in CD4^+^ T cell recovery. Thus, we investigated how the percentage of Tregs within CD4^+^ T cells correlated with CD4^+^ T cell count recovery and other parameters of immune reconstitution in HAART-treated HIV^+^ individuals. Understanding the mechanisms that limit T cell recovery during HAART is essential to help adjust guidelines for HAART initiation, and to prompt investigation of complementary immune therapies that could enhance immune reconstitution.

## Materials and Methods

### Ethics Statement

This study was approved by the institutional review board of the Hospital Joaquim Urbano under the protocol number 168/CES [addenda number 127/12 (NA-DEFI/089-CES)]. Study subjects gave written, informed consent prior to their participation.

### Study population

A cross-sectional study was performed with 53 HIV^+^ individuals recruited from Hospital Joaquim Urbano, Porto, Portugal (43±8 years old, range 31 to 58 years old; 79% were males) and 51 healthy individuals recruited from the same hospital and from the Life and Health Sciences Research Institute, Braga, Portugal (39±9 years old, range 27 to 56 years old; 49% were males). Inclusion criteria for HIV^+^ individuals were: infection with HIV-1; receiving HAART for at least 1 year; being regular on HAART compliance (with no history of irregular compliance in the past); plasma viral loads ≤50 copies HIV RNA/mL; and baseline CD4^+^ T cell counts ≤500 cells/µL. Information regarding patient's gender, hepatitis C virus (HCV) co-infection, HAART compliance, baseline CD4^+^ T cell counts, Nadir value and actual CD4^+^ T cell counts was collected by patients' physician. CD4^+^ T cell counts were obtained by a reference laboratory. CD4^+^ T cell count progression for each individual were calculated by subtracting the baseline CD4^+^ T cell counts (immediately before HAART initiation) from the actual CD4^+^ T cell counts. The CD4^+^ T cell slopes (b1) for each individual during the first 12 months of HAART were calculated by the least square estimation method using MO Excel (CD4^+^ T cell count = b0+b1×time; b0 being the CD4+ T cell counts at 0 months of HAART); analysis was restricted to those subjects who had at least three CD4^+^ T cell measurements during the first year after HAART initiation.

### Flow cytometry

All samples were processed for flow cytometric analysis on the day the blood was collected. To stain for cell surface molecules 100 µL of whole blood were incubated with a defined set of antibodies for 15 min at room temperature, followed by 15 min with FACS Lysis Buffer (BD Biosciences), washed and acquired. To determine the expression of the intracellular marker FOXP3, 2 million peripheral blood mononuclear cells (PBMCs), obtained from heparinized blood by Histopaque 1077 (Sigma-Aldrich) gradient centrifugation, were stained for cell surface markers for 20 min, washed, fixed, permeabilized and stained using the FOXP3 Staining Buffer Set (eBiosciences). Antibodies used were anti-CD4 (clone RPA-T4; BD Biosciences), anti-CD3 (clone OKT3 or UCHT1), anti-CD45RO (clone UCHL1), anti-HLA-DR (clone L243), anti-CD127 (clone PHCD127), anti-CD25 (clone BC96, all from Biolegend) and anti-FOXP3 (clone PCH101, eBiosciences). Optimal concentration was determined for each antibody by testing serial dilutions. All samples were acquired on a BD LSR II flow cytometer using FACS DIVA software (Becton and Dickinson, NJ, USA) and data were analysed using FlowJo Software (Tree Star, OR, USA).

### Statistical analysis

The normality assumption for parametric tests was tested using the Kolmogorov-Smirnov test (with Dallal-Wilkinson-Lilliefor Significance Correction); since the Tregs percentages from HIV^+^ individuals did not follow a normal distribution all tests applied were non-parametric. Groups' medians, variances and proportions were compared using the Mann-Whitney, Levene's and Chi-square tests, respectively. Spearman's rank correlation coefficient was performed to assess the correlation between two variables. P-values less than 0.05 were considered statistically significant.

## Results and Discussion

### Heterogeneous distribution of Tregs percentages among HIV^+^ individuals under HAART

**Figure 1:**
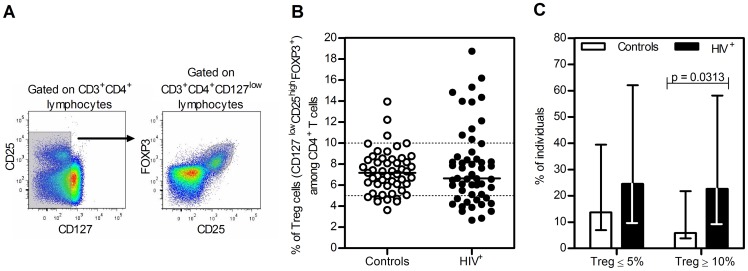
High variability of Tregs percentages in HIV^+^ individuals. **A.** Representative dot plots from an HIV^+^ individual illustrating the gating strategy for Tregs analysis. Lymphocytes (selected according to FSC and SSC) were gated on CD3^+^CD4^+^ cells, on low or no expression of CD127 and on the expression of FOXP3 and high levels of CD25. **B.** Tregs percentages amongst CD4^+^ T cells in control and HIV^+^ individuals. Each dot represents a single individual and the lines the median Tregs percentages within CD4^+^ T cells. Dashed lines represent the range of Tregs percentages among total CD4^+^ T cells described for healthy individuals (between 5% and 10%) [51]. **C.** Percentage of individuals with Tregs ≤5% and ≥10% in controls and HIV^+^ individuals. P-value for the comparison of these proportions (Chi-square test) and the 95% confidence interval are depicted.

Human Tregs were first identified on the basis of their high-level expression of CD25 (the IL-2Rα chain) [40], [41], [42] and subsequently by the expression of the Forkhead-box transcription factor FOXP3 [43], [44]. However, further human studies have shown that activated CD4^+^ T cells also up-regulate the expression of CD25 and can transiently express FOXP3 [45], [46]. More recently, it was shown that Tregs express low levels of CD127 (the IL-7Rα chain), and therefore this molecule is considered useful as an additional marker to identify this population [47], [48], [49], [50]. Even though the utility of these, and other putative Tregs markers, is still debated, they currently represent the best available markers to identify this cell subset. With this in mind, we chose to identify Tregs within CD4^+^ T cells (CD3^+^CD4^+^) as the CD127^low^CD25^high^FOXP3^+^ population (Figure 1A).

Using this gating strategy to define Tregs the vast majority of our control individuals (75%) featured Tregs percentages between 5% and 10% (Figure 1B), frequencies similar to those reported by other groups using the same markers and gating strategy [51]. By comparing the median Tregs percentages no differences were observed between the HIV^+^ and control individuals (p = 0.8250, Mann-Whitney test; Figure 1B). However, the range of Tregs percentages observed was higher amongst HIV^+^ individuals (p = 0.0020, Levene's test; Figure 1B). It was interesting also to note that the number of individuals with Tregs percentages ≥10% was significantly higher in the HIV cohort as compared to control (p = 0.0313, Chi-Square test; Figure 1C).

As studies of HAART-treated HIV^+^ individuals have yielded conflicting data regarding Tregs percentages [22], [23], [35], [36], [37], [38], [39], we sought to understand if other variables could be influencing this parameter. We found that neither individual's age, number of years in therapy, overall immune activation or infection by the hepatitis C virus (HCV) impacted upon Tregs percentage in our HIV population (Figure S1).

Overall, whilst there were no differences in the median percentage of Tregs in HIV^+^ and control individuals, there was a wider distribution of Tregs percentages among HIV^+^ individuals, ranging from 3% to 19%. Furthermore, Tregs frequencies were not related to age, number of years of treatment, overall immune activation or infection by HCV (Figure S1).

### The majority of HIV^+^ individuals with high Tregs percentages featured low Nadir value and incomplete CD4^+^ T cell recovery

**Figure 2:**
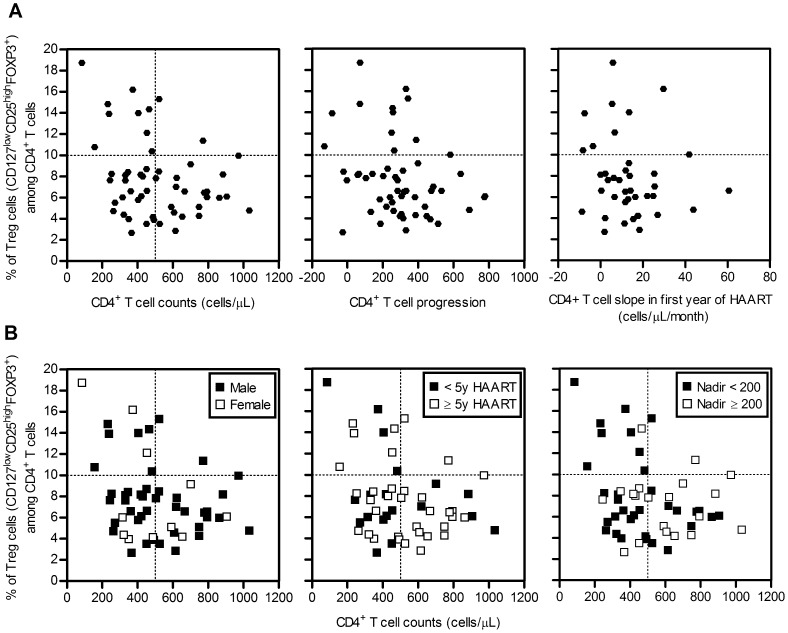
HIV^+^ individuals with high Tregs percentages feature low CD4^+^ T cell counts and Nadir values. **A.** Percentages of Tregs amongst HIV^+^ individuals were plotted against CD4^+^ T cell counts, CD4^+^ T cell progression and CD4^+^ T cell slope (for the first year of HAART). Each dot represents a single individual. **B.** Tregs percentages were plotted against CD4^+^ T cell counts in HIV^+^ individuals grouped by gender) number of years under HAART and the Nadir value.

To evaluate whether distinct Tregs percentages were related to different degrees of CD4^+^ T cell recovery amongst HAART-treated HIV^+^ individuals, we assessed potential correlations between distinct parameters to evaluate CD4^+^ T cell reconstitution and Tregs percentages. As shown in Figure 2A, we found no correlation between Tregs percentages and CD4^+^ T cell counts, CD4^+^ T cell progression or CD4^+^ T cell slope (R = −0.2130 and p = 0.1270; R = −0.2409 and p = 0.0823; R = −0.1836 and p = 0.2633, respectively, Spearman's correlation). While there is no clear correlation between the CD4^+^ T cell reconstitution and Tregs percentage, as previously reported by others [37], [39], it is interesting to note that amongst HIV^+^ individuals with high Tregs proportions (≥10%) the majority featured low CD4^+^ T cell numbers (<500 cells/µL), low CD4^+^ T cell recovery and low CD4^+^ T cell slope (Figure 2A).

To further dissect the link between low CD4^+^ T cell counts and high Tregs percentages in HIV^+^ individuals we re-assessed the relationship between these two variables taking into account the gender, number of years on treatment and the Nadir value, all of which are factors known to influence immune reconstitution [3], [4], [5], [15], [52], [53]. No clear correlations were observed between the Tregs percentages and CD4^+^ T cell counts when patient's gender (R = −0.2480 and p = 0.1090 for males; R = −0.2480 and p = 0.4920 for females, Spearman's correlation), years of therapy (R = −0.0772 and p = 0.7534 for <5 y HAART; R = −0.2598 and p = 0.1379 for ≥5 y HAART, Spearman's correlation) and the Nadir value (R = −0.07719 and p = 0.0648 for <200 cells/µL, R = 0.0,0963 and p = 0.6893 for ≥200 cells/µL, Spearman's correlation) were taken in consideration (Figure 2B).

**Figure 3:**
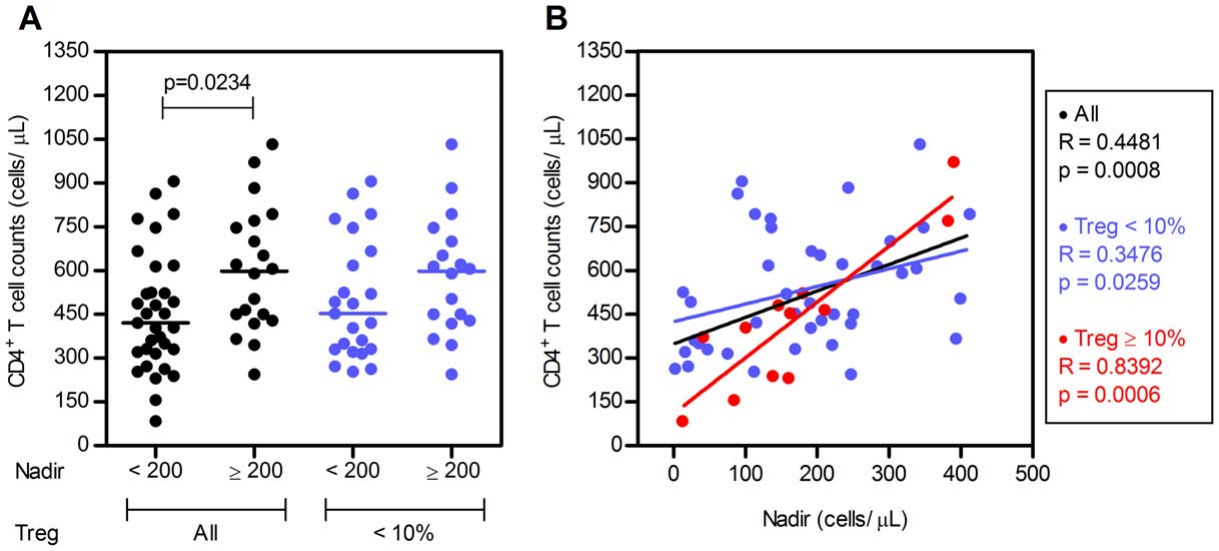
Individuals with high Tregs percentages feature the strongest correlation between CD4^+^ counts and Nadir values. **A.** HIV^+^ individuals were subdivided according to their Nadir counts (<200 and ≥200 cells/µL) and grouped according to Tregs percentages (all individuals and individuals with <10% Tregs) and the CD4^+^ T cell counts are shown for each group. Each dot represents a single individual and the line the median value for each group. P-value for the comparison of the CD4^+^ T cell counts is indicated (Mann-Whitney test). **B.** Scatter plots illustrating the correlation between Nadir value and CD4^+^ T cell counts during HAART. Each dot represents a single individual. P-value for the correlation between CD4^+^ T cell counts and the Nadir value is shown (Spearman's correlation).

Of notice, we observed that almost all individuals with Tregs percentage ≥10% and CD4^+^ T cell counts ≤500 cells/µL had Nadir values <200 cells/µL (Figure 2B). Since the Nadir value has been considered one of the key factors influencing immune reconstitution [3], [4], [5], we considered relevant to further explore this association. To do so we analysed CD4^+^ T cell counts in HIV^+^ individuals divided on the basis of their Nadir values (<200 and ≥200 cells/µL) and subdivided according to their Tregs frequency (<10%, Figure 3A). As previously reported [3], [4], [5], we observed that individuals with low Nadir values (<200 cells/µL) had lower CD4^+^ T cell counts upon treatment (p = 0.0234, Mann-Whitney test, Figure 3A). Interestingly, this difference lost statistical significance when only those individuals with Tregs percentages <10% were analysed (p = 0.1934 by Mann-Whitney test, Figure 3A).To address the influence of Tregs percentages on the well-established correlation between CD4^+^ T cell recovery and Nadir value [3], [4], [5], a correlation between these two variables separating the individuals according to the Tregs percentages was performed. As shown previously by others we observed that CD4^+^ T cell counts positively correlate with the Nadir value when all HIV^+^ individuals are considered (R = 0.4481 and p = 0.0008, Spearman's correlation; Figure 3B). Of interest this correlation was much stronger when only those individuals with ≥10% Tregs were taken into account (R = 0.8392 and p = 0.0006 by Spearman's correlation; Figure 3B) indicating that CD4^+^ T cell recovery of individuals with low Nadir is hampered by high proportions of Tregs.

Results reported by Gaardbo *et al.* [36], who analysed a correlation between CD4^+^ T cell counts and Tregs percentages in patients sub-divided according to baseline CD4^+^ T cell counts (< and >200 cells/µL), did not find any differences. Their analysis focused on HIV^+^ patients with over 2 years of treatment. Since we have observed that time in treatment seems to have no impact on the CD4^+^ T cell counts *vs.* Tregs proportion relation, the discrepancy is most likely due to the use of different markers to define Tregs or to the fact that they used baseline CD4^+^ counts instead of Nadir values.

### Strong correlation between Tregs percentages and CD4^+^ T cell counts progression in immunologically non-responders HIV^+^ individuals

**Figure 4:**
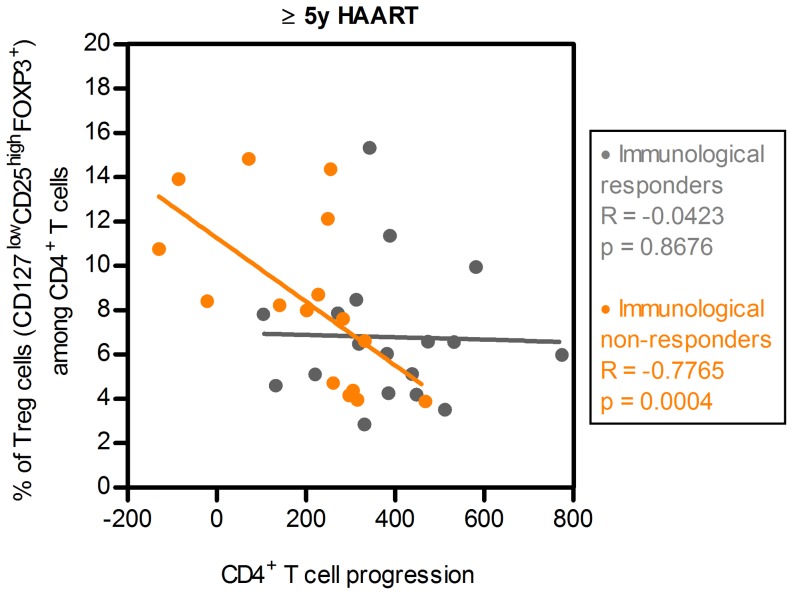
Immunological non-responders present a strong correlation between Tregs percentages and CD4^+^ T cell progression. Scatter plot illustrating the correlation between Tregs percentages and CD4^+^ T cell progression. Patients were selected on the basis of being under HAART for at least 5 years and whose actual CD4^+^ T cell counts are <500 cells/µL or >500 cells/µL. Each dot represents a single individual.

While the observation that some individuals are unable to reconstitute the CD4^+^ T cell numbers to normal values, even after several years of therapy and suppression of viral replication, there is still a lack of consensus on the definition of immunological non-responder individuals [7]. The most well accepted definition for immunological non-responders patients are the ones whose CD4^+^ T cell counts remained below a threshold (from 350 to 500 cells/µL) after a variable period of time of treatment (from 4 to 7 years) [3], [4], [5]. Considering as immunological non-responders the individuals under regular HAART for at least 5 years and whose CD4^+^ T cell counts were <500 cells/µL (14 out of 53 individuals in our population), we observed a strong correlation between Tregs percentages and CD4^+^ T cell progression (R = −0.7765 and p = 0.0004, Spearman's correlation, Figure 4), which strengthens the observed association between high Tregs percentage and poor CD4^+^ T cell reconstitution.

The biological processes that could lead to different Tregs percentages in HIV^+^ individuals remain unclear. Whilst some studies have shown that Tregs are permissive to HIV infection *in vitro* [19], [20], which could lead to the depletion of this subset in HIV^+^ individuals, others have shown that HIV selectively promotes Tregs survival by reducing apoptosis levels in this subset, and thereby increasing their proportion within the CD4^+^ T cell pool as a whole [21]. Several hypotheses have been put forward in order to explain the observed differences in Tregs percentages, such as increased Tregs thymopoiesis [37] or even conversion of conventional CD4^+^ T cells to Tregs [54], [55]. Further studies are still needed in order to fully understand Tregs kinetics during immune reconstitution in HAART-treated HIV^+^ individuals.

Altogether, our data show that high proportions of Tregs during treatment in individuals who reached low Nadir values (<200 CD4^+^ T cells/µL) have a synergistic/cumulative negative association with incomplete CD4^+^ T cell recovery. While the clear association of high percentage of Tregs with low Nadir and incomplete reconstitution would suggest a potential negative impact of Tregs on CD4^+^ T cell recovery, it cannot be excluded the possibility that Tregs, in individuals with incomplete immune reconstitution, play an important role preventing excessive expansions of oligoclonal populations. This could be of importance to better decide when to start treatment. If during the course of HIV infection an individual already has a high Tregs percentage, our results would support the need for HAART initiation even if CD4^+^ T cells counts remain relatively high. Further studies involving longitudinal follow up are needed in order to fully understand how the high percentage of Tregs abrogates the CD4^+^ T cells recovery. Moreover, these studies could also help defining the parameters that synergise with high Tregs frequencies and low Nadir for sub-optimal T cell recovery.

## Supporting Information

Figure S1 **Tregs percentages are not affected by HCV infection, age, time in treatment or immune activation.** **A.** Tregs percentages were compared in HIV^+^ individuals when divided according to co-infection with HCV (p = 0.1250, Mann-Whitney test). **B.** Relationship between the Tregs percentages and age (left panel; R = −0.1520, p = 0.2773; Spearman's correlation), years in therapy (middle panel; R = −0.0682, p = 0.6277; Spearman's correlation) and overall immune activation (right panel; R = 0.1400, p = 0.3660; Spearman's correlation). Each dot represents a single individual.
(TIF)
